# New Matching Method for Accelerometers in Gravity Gradiometer

**DOI:** 10.3390/s17081710

**Published:** 2017-07-25

**Authors:** Hongwei Wei, Meiping Wu, Juliang Cao

**Affiliations:** College of Mechatronics and Automation, National University of Defense Technology, Changsha 410073, China; weihongweier@nudt.edu.cn (H.W.); jlcao@nudt.edu.cn (J.C.)

**Keywords:** matching method, gravity gradiometer, configuration

## Abstract

The gravity gradiometer is widely used in mineral prospecting, including in the exploration of mineral, oil and gas deposits. The mismatch of accelerometers adversely affects the measuring precision of rotating accelerometer-based gravity gradiometers. Several strategies have been investigated to address the imbalance of accelerometers in gradiometers. These strategies, however, complicate gradiometer structures because feedback loops and re-designed accelerometers are needed in these strategies. In this paper, we present a novel matching method, which is based on a new configuration of accelerometers in a gravity gradiometer. In the new configuration, an angle was introduced between the measurement direction of the accelerometer and the spin direction. With the introduced angle, accelerometers could measure the centrifugal acceleration generated by the rotating disc. Matching was realized by updating the scale factors of the accelerometers with the help of centrifugal acceleration. Further simulation computations showed that after adopting the new matching method, signal-to-noise ratio improved from −41 dB to 22 dB. Compared with other matching methods, our method is more flexible and costs less. The matching accuracy of this new method is similar to that of other methods. Our method provides a new idea for matching methods in gravity gradiometer measurement.

## 1. Introduction

Spatial variations in gravity are reflected by the gravity gradient tensor, which is the second-order spatial derivative of gravitational potential. Compared with traditional gravity signals, the gravity gradient includes signals with smaller spatial extend, which are used for the determination of the short-to-medium wavelengths of the gravity field [1]. Gravity gradient signals can provide the small-scale features of sources, such as oil, gas and mineral resources buried in the ground [2]. As a high-accuracy, high-resolution signal of the gravity field, the gravity gradient has an important role in hydrocarbon or mineral exploration, geophysics and inertial navigation [2,3,4,5,6,7,8,9,10,11]. With the Euler deconvolution as an example, more detailed information and more accurate results could be obtained through gravity gradient data [6]. In addition, gravity gradient data also make a contribution to calculate the depths of particular sources directly [7]. For example, salt keels in the Gulf of Mexico have been mapped using marine gravity gradiometry combined with 3D seismic data. Airborne gravity gradiometry has been used in kimberlite exploration in Canada [12]. As an important remote sensing technique, gradient data also provided a new way to map the GaraDjebilet iron ore region, which is one of the most important regions in southwestern Africa. Furthermore, the assistant navigation for Inertial Measuring Units (IMU) is another approach to use high-resolution gravity gradient data [10,11,13].

Many types of high-precision gravity gradient instruments, for obtaining gravity gradient data, have been developed based on different principles. The first gravity gradiometer is the Loránd Eötvös’ torsion balance, which was designed by Hungarian physicist Baron von Eötvös. Although it is useful in oil and gas exploration, the use of this bulky terrestrial gradiometer has been practically phased out because of its low measurement efficiency [14,15]. Since the 1970s, many novel gravity gradiometers have been developed, such as:

- Gravity Field and Steady-State Ocean Circulation Explorer (GOCE), which is a satellite gravity gradient instrument launched by the European Space Agency. It has three pairs of ultra-sensitive accelerometers mounted at the end-points of three orthogonal axes. The noise of these accelerometers is approximately 1–11 pm $s^{-2}/\sqrt{\mathrm{Hz}}$ [14,16].
- Full Tensor Gradiometer (FTG; based on rotating accelerometers), which was designed and manufactured by Lockheed Martin, is now widely used in hydrocarbon and mineral exploration [17].
- Airborne gravity gradiometer (Falcon Group Inc., Bloomfield Hills, MI, USA) (also based on a rotating accelerometer), which is a partial tensor system of BHP Billiton [18].
- Exploration Gravity Gradiometer, an ARKeX gradiometer (ARKeX, Cambridge, UK), was specifically designed for high dynamic survey environment. It has a target sensitivity resolution of $1E\left(1E=10^{-9}s^{-2}\right)/\sqrt{\mathrm{Hz}}$ [19].
- High-Definition Airborne Gravity Gradiometer (Gedex Inc., Mississauga, ON, Canada) will be used in airborne geophysical surveys and the exploration of mineral, oil and gas deposits. This gradiometer has a pair of balance beams to measure the gravity gradient. Each balance beam is centered on a pivot spring. Its target sensitivity resolution is $1E/\sqrt{\mathrm{Hz}}$ in the bandwidth range of 0.001 Hz to 1 Hz [20].
- VK-1, an airborne gravity gradiometer, was developed by the University of Western Australia (UWA) based on two crosswise bars. The performance objective of the instrument is $1E/\sqrt{\mathrm{Hz}}$ [21,22].
- GREMLIT (French Aerospace Lab, Palaiseau, France), a compact planar gravity gradiometer based on four ultra-sensitive electrostatic planar accelerometers (inherited from technologies specifically developed for the GOCE and Gravity Recovery and Climate Experiment missions), was designed by the Onera-French Aerospace Lab and is used for airborne surveys [14].
- AI (Earth Observation Programmes, European Space Agency, Noordwijk, the Netherlands) are gravity gradiometers that are based on cold-atom interferometry-based accelerometers and used to measure all diagonal elements of the gravity gradient tensor. The accuracy of the cold-atom interferometry-based accelerometer is approximately 10^−12^ m/s^2^. These gradiometers provide absolute measurements and do not require calibration prior to use. The European Space Agency has proposed a space-borne gravity gradiometer concept based on cold-atom interferometers; the proposed gradiometer has a sensitivity of $3.5\mathrm{mE}/\sqrt{\mathrm{Hz}}$ [23,24].

Among these gradiometers, FTG and FALCON are the only two measuring instruments that have passed the flight test and achieved acceptable sensitivity in the field surveys with high-resolution [25]. FTG and FALCON airborne gravity gradiometer survey systems have been used in iron ore exploration in the Bau Mine Site, Quadrilatero Ferrifero, State of Minas Gerais, Brazil [5]. Thus, unless explicitly stated in the rest of this article, “gravity gradiometer” refers to rotating accelerometer-based gradiometers, such as FTG and FALCON.

**Figure 1:**
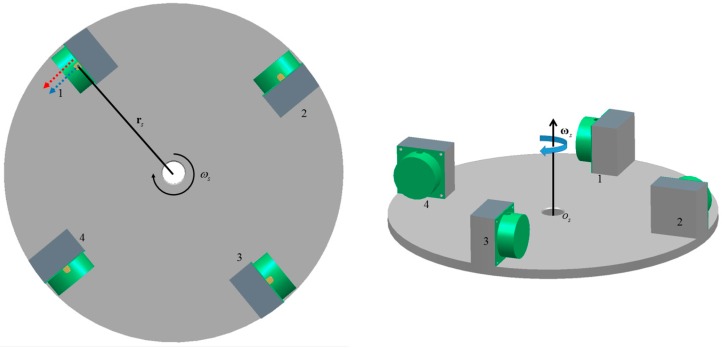
Schematic of gravity gradient instrument (GGI), which consists of four accelerometers (1, 2, 3, 4). The red arrow represents the direction of the sensitive axis of the accelerometer and the blue arrow is the tangential direction; $r_{s}$ is the radius of the disc; and $\omega_{s}$ is the angular velocity of the disc.

The rotating accelerometer gravity gradiometer utilizes differences between the outputs of linear accelerometer pairs to extract gravity gradient signals. The gravity gradient instrument (GGI), which is a disc mounted with accelerometers (Figure 1), is the core element of FTG and FALCON. Two complementary pairs of accelerometers are mounted with equal spacing around the circumference of a disc. The sensitivity axes of each accelerometer and the tangent line of the disc are parallel. The disk rotates around its central axis.

The configuration of accelerometers in GGI is shown in Figure 1. With this classical configuration, common mode accelerations, which are generated by the motion of the carrier (such as aircraft, vehicle and ships) and the external environment, could ideally be canceled. The common-mode rejection gives GGI immunity to kinematic accelerations. Furthermore, the rotation of the turntable is needed to mitigate for the erratic shift of the outputs of the sensors. With the rotation, the gravitational gradient signal is modulated to twice the frequency of the rotation through the rotation of the turntable, making the gravitational gradient signal and the noise at different frequency bands, and aiding for the extraction of the gravitational gradient signal. 

However, the common-mode rejection performance of GGI depends on the premise that opposite accelerometers are precisely matched. This premise is not always satisfied. When two paired accelerometers do not match perfectly, the common mode acceleration cannot be completely suppressed. Thus, the signal-to-noise ratio (SNR) is drastically reduced, making signal extraction difficult. Therefore, several strategies have been investigated to address the imbalance of accelerometers. For instance, the electromagnetic feedback trimming technique is used in Bell’s GGI; the accelerometers are matched by adjusting the sensitivity of the accelerometer through a trim coil and a feedback loop. A proper current determined by the feedback loop is injected into the trim coil and the scale factor balances are then obtained [26,27]. Tu et al. developed an electrostatic servo-controlled trimming technique to match accelerometers. A redesigned accelerometer and electrostatic servo-controlled actuator was used in this technique, and the balances between a pair of accelerometers were realized by directly trimming the electrostatic bias voltage [25]. This problem also exists in GOCE. Two steps are taken to eliminate the influence of accelerometer imperfections on the GOCE gradiometer. The first step is to correct the non-linear terms in accelerometers by slightly changing the proof mass position. In the second step, 72 parameters, which describe the mismatch between accelerometers of the gradiometer are determined. These parameters are retrieved from the data obtained from the shaking of the spacecraft [28]. All these technologies are aimed to balance the scale factor between a pair of accelerometers using hardware method. Although, these techniques have contributed to gravity gradient measurement, the former two methods require the real-time adjustment of accelerometers, thus considerably complicating the measurement of the gravity gradient. For example, the electromagnetic actuator in the feedback control loop and the extra trimming assembly are necessary in the electromagnetic feedback trimming technique. Three external, remotely balanced loops and redesigned accelerometers are needed in the electrostatic servo-controlled trimming technique [25,26,27]. The calibration method of GOCE works well, but is unsuitable for the rotating accelerometer-based gradiometer because the structures and working environment of the two gradiometers are different.

In this paper, we propose a new matching method for general quartz-flexure capacitive accelerometers. Our proposed method is based on GGI using a new configuration. First, we proposed a new configuration of GGI to allow the accelerometers to directly measure centrifugal acceleration caused by disc rotation. Second, we present a new matching method based on the new configuration. We designed a simulation test to analyze the validity of the new method. Finally, we provide our conclusions and suggestions for future works on accelerometer matching. The new method will help us reduce the instability caused by the drift of time-varying scale factors and increase the precision of measurement.

This paper is structured as follows: the basic principle of gravity gradient measurement is introduced in the second section. In the third section, the new GGI configuration and the new matching method are presented. The simulation test for the efficiency of the new method is discussed. The discussion and conclusion are given in the final section.

## 2. Basic Principle of Gravity Gradient Measurement

Newton’s Second Law of Motion is the theoretical foundation for the rotating accelerometer-based gravity gradiometer:

(1) $${\frac{d^{2}r}{dt^{2}}|}_{i}=a+g$$

where ${\frac{d^{2}r}{dt^{2}}|}_{i}$ is the acceleration of the carrier, **a** is the specific acceleration measurable by the accelerometer, and **g** is the acceleration vector caused by gravity at the measuring point.

**Figure 2:**
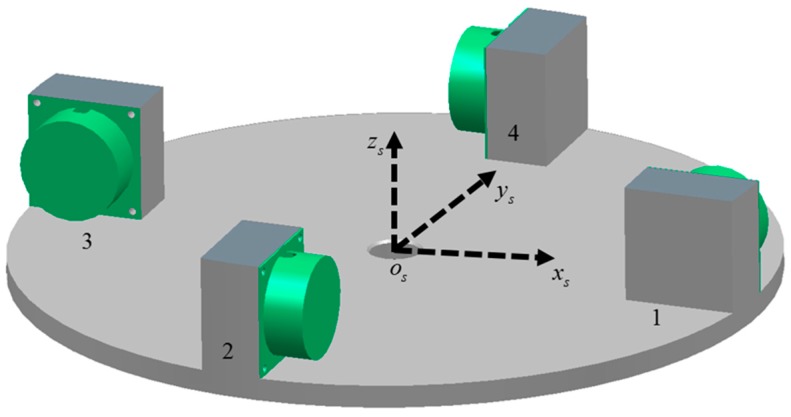
Instrument frame fixed to the carrier. The origin of the instrument frame is located at the center of the disc.

The basic principle of gravity gradient measurement will be analyzed in the instrument frame, which is a moving frame fixed to the carrier as shown in Figure 2. The instrument frame has its origin at the center of the turntable (point $o_{s}$). Non-rotating axes with respect to the carrier are defined by $o_{s}x_{s},o_{s}y_{s},o_{s}z_{s}$, with $o_{s}z_{s}$ coincident with the central axis of the disc. $o_{s}x_{s}$ lies along the direction from $o_{s}$ to the initial position of the accelerometer 1. 

Projecting Equation (1) into the instrument frame:

(2) $$a_{i}=a_{m}-g_{i}$$

where $a_{i}\left(i\in \left\{1,2,3,4\right\}\right)$ is the specific acceleration where accelerometer i is mounted, $a_{m}$ is the vector of non-gravitational acceleration caused by motion and $g_{i}$ represents the gravitational acceleration where accelerometer i is mounted. For all accelerometers, $a_{m}$ is the same because these accelerometers are mounted on the same turntable.

The direction of the sensitive axis of the accelerometers has the following relations:

(3) $$d_{1}=-d_{3},\;d_{2}=-d_{4}$$

where $d_{i}\left(i\in \left\{1,2,3,4\right\}\right)$ is the direction of the sensitive axis of the accelerometer i.

Accordingly, we have:

(4) $$\begin{array}{l} a_{1}=a_{1}\times d_{1}=a_{m}\times d_{1}-g_{1}\times d_{1}\\ a_{2}=a_{2}\times d_{2}=a_{m}\times d_{2}-g_{2}\times d_{2}\\ a_{3}=a_{3}\times d_{3}=-a_{m}\times d_{1}+g_{3}\times d_{1}\\ a_{4}=a_{4}\times d_{4}=-a_{m}\times d_{2}+g_{4}\times d_{2} \end{array}$$

where $a_{i}\left(i\in \left\{1,2,3,4\right\}\right)$ is the reading of accelerometer i.

Adding opposite accelerometer readings, such as those of accelerometers 1 and 3:

(5) $$a_{1}+a_{3}=\left(a_{m}\times d_{1}-a_{m}\times d_{1}\right)+\left(g_{3}-g_{1}\right)\times d_{1}$$

The first term on the right side of Equation (5) is equal to zero, indicating that non-gravitational accelerations are canceled, this is the basic principle of the common mode rejection. When the accelerometers do not match, this term is not zero. Therefore, Equation (5) could be used to control how well the common mode rejection works.

Using the first-order Taylor expansion of $g_{1}$ and $g_{3}$ at the center of the turntable, and taking the preceding two items, we obtain the following:

(6) $$g_{3}-g_{1}=\Gamma \times dr$$

where Γ is the gravity gradient tensor and dr is the vector from accelerometer 1 to accelerometer 3.

Substituting Equation (6) into Equation (5), will yield the following relation [29]:

(7) $$a_{1}+a_{3}=\Gamma_{xj}dr_{j}\left(j\in \left\{x,y,z\right\}\right)$$

where $a_{1}$ and $a_{3}$ are the outputs of accelerometers 1 and 3, respectively. $\Gamma_{xj}$ is the gravity gradient tensor; and $dr_{j}$ is the distance vector between the two accelerometers.

In case of a horizontal disk, $dr_{j}$ can be expressed in the instrument frame as:

(8) $$\begin{array}{l} dr_{x}=2r_{s}\cos\alpha \\ dr_{y}=2r_{s}\sin\alpha \\ dr_{z}=0 \end{array}$$

where α is the angle between the x-axis and accelerometer 1 in the instrument frame. α can be calculated by $\alpha =\omega_{s}t$ and $r_{s}$ represents the radius of the disc.

Substituting Equations (3)–(6) and (8) into Equation (7) [18], it then follows that for the accelerometer difference $a_{1}+a_{3}$ and $a_{2}+a_{4}$ on a horizontal disc, rotating with angular speed $\omega_{s}$ at time t that

(9) $$\left(a_{1}+a_{3}\right)-\left(a_{2}+a_{4}\right)=4r_{s}\Gamma_{xy}\cos2\omega_{s}t+2r_{s}\left(\Gamma_{xx}-\Gamma_{yy}\right)\sin2\omega_{s}t$$

Equation (9) indicates that the $\Gamma_{xy}$ and $\left(\Gamma_{xx}-\Gamma_{yy}\right)$ components of the gravity gradient tensor could be obtained by demodulating $\left(a_{1}+a_{3}\right)-\left(a_{2}+a_{4}\right)$ at a frequency of $2\omega_{s}t$.

## 3. New Configuration and Matching Method

As mentioned above, for the classical configuration of accelerometers in GGI (Figure 1), the sensitivity axis of each accelerometer and the tangent line of the disc are parallel, and the outputs of accelerometers are unaffected by the angular velocity of the disc. Therefore, we proposed a new configuration to match accelerometers based on the centrifugal acceleration caused by the angular velocity of the disc. 

### 3.1. New Configuration

**Figure 3:**
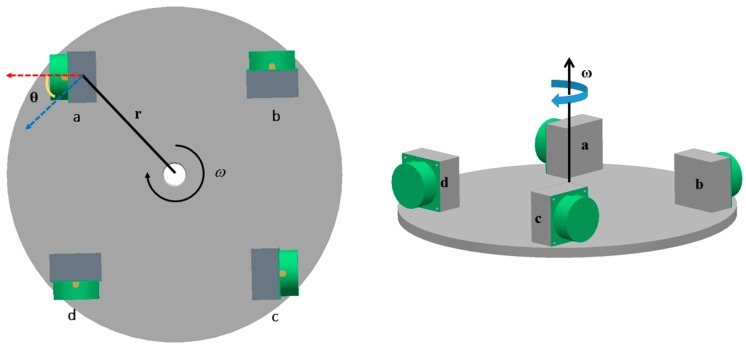
New GGI configuration. A fixed angle lies between the measurement direction (red line) of accelerometer and the spin direction (blue line). The rotational speed and radius of the turntable are denoted by ω and r, respectively.

The new configuration consists of two opposing pairs of accelerometers (Figure 3).

The direction of the sensitive axis is indicated by a red line, and the tangential direction is indicated by a blue line, as shown in Figure 3. The fixed angle between these two axes is denoted by θ. Thus, the centrifugal acceleration caused by the rotation could be measured by the accelerometer as $r\omega^{2}\sin\theta$, and could be used to match accelerometers.

### 3.2. Accelerometer Model

An absolute linear relationship between the input and output of the accelerometer does not exist in practical measurement. A simple model generally used by an Inertial Measurement Unit (IMU) is expressed as follows:

(10) $$N=k_{1}\left(a+b+v\right)$$

where N is the accelerometer reading, that is, the number of pulses; a is the true value of the specific force (which contains the gravitational gradient signal); $k_{1}$ is the linear scale factor; and b and v are the bias and the noise of the accelerometer, respectively.

However, nonlinear scale factors are inherent in the output of high-precision accelerometers [30,31]. Furthermore, the ignored errors caused by the nonlinear scale factors affect the quality of inertial sensors [32]. Given that the gravity gradient signal is very weak, a high-precision accelerometer model is necessary. Therefore, nonlinear scale factors should be introduced to the sensor model.

The quadratic term is the main component in scale factors; other components are all negligible [33]. To simplify the model, the other second- and higher-order terms are omitted. The model with a quadratic factor is derived as follows:

(11) $$N=k_{1}\left(a+k_{2}a^{2}+b+v\right)$$

where $k_{2}$ is the quadratic factor.

Taking points a and c in Figure 3 into account, and combining the accelerometer model described in Equation (11), the outputs of accelerometers a and c can be represented as

(12) $$N_{a}=k_{1a}\left(a_{a}+k_{2a}a_{a}^{2}+b_{a}+v_{a}\right)$$

and

(13) $$N_{c}=k_{1c}\left(a_{c}+k_{2c}a_{c}^{2}+b_{c}+v_{c}\right)$$

where $N_{i}\left(i\in \left\{a,c\right\}\right)$ is the output of accelerometer i; $a_{i}\left(i\in \left\{a,c\right\}\right)$ is the true value of the specific force at i; $k_{1i}\left(i\in \left\{a,c\right\}\right)$ is the linear scale factor; $k_{2i}\left(i\in \left\{a,c\right\}\right)$ is the quadratic factor; and $b_{i}\left(i\in \left\{a,c\right\}\right)$ and $v_{i}\left(i\in \left\{a,c\right\}\right)$ are the bias and the noise of the accelerometer i, respectively.

### 3.3. Principle of the New Matching Method

The new matching method for accelerometers a and c is realized by adjusting the parameters $k_{1i},k_{2i},b_{i}\left(i\in \left\{a,c\right\}\right)$ in Equations (12) and (13). 

For the accelerometers configured as in Figure 3, inputs can be divided into two parts: the centrifugal acceleration produced by the disc and others (all accelerations except for the centrifugal acceleration caused by the rotation of the turntable), as denoted by $f_{\omega }$ and $f_{i}\left(i\in \left\{a,c\right\}\right)$, respectively. The other inputs include acceleration produced by gravity and other non-gravitational acceleration. Substituting these inputs into Equations (12) and (13), we obtain:

(14) $$N_{a}=k_{1a}\left(\left(f_{a}+f_{\omega }\right)+k_{2a}{\left(f_{a}+f_{\omega }\right)}^{2}+b_{a}+v_{a}\right)$$

and

(15) $$N_{c}=k_{1c}\left(\left(-f_{c}+f_{\omega }\right)+k_{2c}{\left(-f_{c}+f_{\omega }\right)}^{2}+b_{c}+v_{c}\right)$$

If we slow down the angular velocity of the turntable from ω to $\omega -\Delta \omega_{1}$, the centrifugal acceleration of the turntable will change by $\Delta f_{\omega }$. Correspondingly, the outputs of the accelerometers (denoted by ${\hat{N}}_{a}$ and ${\hat{N}}_{c}$) could be written as

(16) $${\hat{N}}_{a}=k_{1a}\left(\left(f_{a}+f_{\omega }-\Delta f_{\omega }\right)+k_{2a}{\left(f_{a}+f_{\omega }-\Delta f_{\omega }\right)}^{2}+b_{a}+v_{a}\right)$$

(17) $${\hat{N}}_{c}=k_{1c}\left(\left(-f_{c}+f_{\omega }-\Delta f_{\omega }\right)+k_{2c}{\left(-f_{c}+f_{\omega }-\Delta f_{\omega }\right)}^{2}+b_{c}+v_{c}\right)$$

If we turn the angular velocity of the turntable from ω to $\omega +\Delta \omega_{2}$, we obtain the following:

(18) $${\hat{\hat{N}}}_{a}=k_{1a}\left(\left(f_{a}+f_{\omega }+\Delta f_{\omega }\right)+k_{2a}{\left(f_{a}+f_{\omega }+\Delta f_{\omega }\right)}^{2}+b_{a}+v_{a}\right)$$

(19) $${\hat{\hat{N}}}_{c}=k_{1c}\left(\left(-f_{c}+f_{\omega }+\Delta f_{\omega }\right)+k_{2c}{\left(-f_{c}+f_{\omega }+\Delta f_{\omega }\right)}^{2}+b_{c}+v_{c}\right)$$

where ${\hat{\hat{N}}}_{a}$ and ${\hat{\hat{N}}}_{c}$ are outputs of accelerometers after the angular velocity is adjusted.

We calculate the sum of Equations (14) and (15) by the following:

(20) $$\begin{array}{cc} N_{ac} & =N_{a}+N_{c}\\ & =K_{1}f_{a}+K_{2}\left(\delta f+f_{\omega }\right)+K_{3}f_{a}^{2}+K_{3}{\left(\delta f+f_{\omega }\right)}^{2}+2K_{4}f_{a}\left(\delta f+f_{\omega }\right)+K_{5} \end{array}$$

where:

(21) $$\begin{array}{l} K_{1}=k_{a1}-k_{c1}\\ K_{2}=k_{a1}+k_{c1}\\ K_{3}=k_{a1}k_{a2}+k_{c1}k_{c2}\\ K_{4}=k_{a1}k_{a2}-k_{c1}k_{c2}\\ K_{5}=k_{a1}b_{a}+k_{c1}b_{c}\\ \delta f=f_{c}-f_{a} \end{array}$$

and a similar relation holds for Equations (16) and (17), as well as for Equations (18) and (19). That is:

(22) $$\begin{array}{cc} {\hat{N}}_{ac} & ={\hat{N}}_{a}+{\hat{N}}_{c}\\ & =K_{1}f_{a}+K_{2}\left(\delta f+f_{\omega }-\Delta f_{\omega }\right)+K_{3}f_{a}^{2}\\ & +K_{3}{\left(\delta f+f_{\omega }-\Delta f_{\omega }\right)}^{2}+2K_{4}f_{a}\left(\delta f+f_{\omega }-\Delta f_{\omega }\right)+K_{5} \end{array}$$

and

(23) $$\begin{array}{cc} {\hat{\hat{N}}}_{ac} & ={\hat{\hat{N}}}_{a}+{\hat{\hat{N}}}_{c}\\ & =K_{1}f_{a}+K_{2}\left(\delta f+f_{\omega }+\Delta f_{\omega }\right)+K_{3}f_{a}^{2}\\ & +K_{3}{\left(\delta f+f_{\omega }+\Delta f_{\omega }\right)}^{2}+2K_{4}f_{a}\left(\delta f+f_{\omega }+\Delta f_{\omega }\right)+K_{5} \end{array}$$

The variables c,b and e in Equation (21) can be calculated from Equation (20), Equations (22) and (23) as follows:

(24) $$\begin{array}{cc} {\hat{\hat{N}}}_{ac}+{\hat{N}}_{ac}= & 2K_{1}f_{a}+2K_{2}\left(\delta f+f_{\omega }\right)+2K_{3}f_{a}^{2}+2K_{3}{\left(\delta f+f_{\omega }\right)}^{2}\\ & 2K_{3}\Delta f_{\omega }^{2}+4K_{4}f_{a}\left(\delta f+f_{\omega }\right)+2K_{5} \end{array}$$

Then:

(25) $${\hat{\hat{N}}}_{ac}+{\hat{N}}_{ac}-N_{ac}=2K_{3}\Delta f_{\omega }^{2}$$

Equation (25) indicates that c could be obtained by:

(26) $$K_{3}=\frac{{\hat{\hat{N}}}_{ac}+{\hat{N}}_{ac}-N_{ac}}{2\Delta f_{\omega }^{2}}$$

We can obtain b and e according to the following Equations:

(27) $$K_{2}=\frac{{\hat{\hat{N}}}_{ac}-{\hat{N}}_{ac}-4K_{3}F\Delta f_{\omega }}{2\Delta f_{\omega }}$$

and

(28) $$K_{5}=N_{ac}-K_{2}F-K_{3}F^{2}$$

where F is the centrifugal acceleration $r\omega^{2}\sin\theta$, which is measured by the accelerometer.

In the above process, consider that the parameters such as $\omega ,\Delta \omega_{1},\Delta \omega_{2}$ are known. Furthermore, the data of $N_{A},N_{C},{\hat{N}}_{A},{\hat{N}}_{C},{\hat{\hat{N}}}_{A},{\hat{\hat{N}}}_{C}$ are stored by data-acquisition systems. Thus, the mismatch between accelerometers a and c, namely, the differential mode parameters $k_{1c}-k_{1a},k_{2c}-k_{2a},b_{c}-b_{a}$ and drifts of them can be calculated. The matching for accelerometers would be realized by mismatch compensation, i.e., adjusting the parameters of one accelerometer in one pair. The detailed steps of the new matching method are introduced in later sections of this paper.

### 3.4. Matching Algorithm

**Figure 4:**
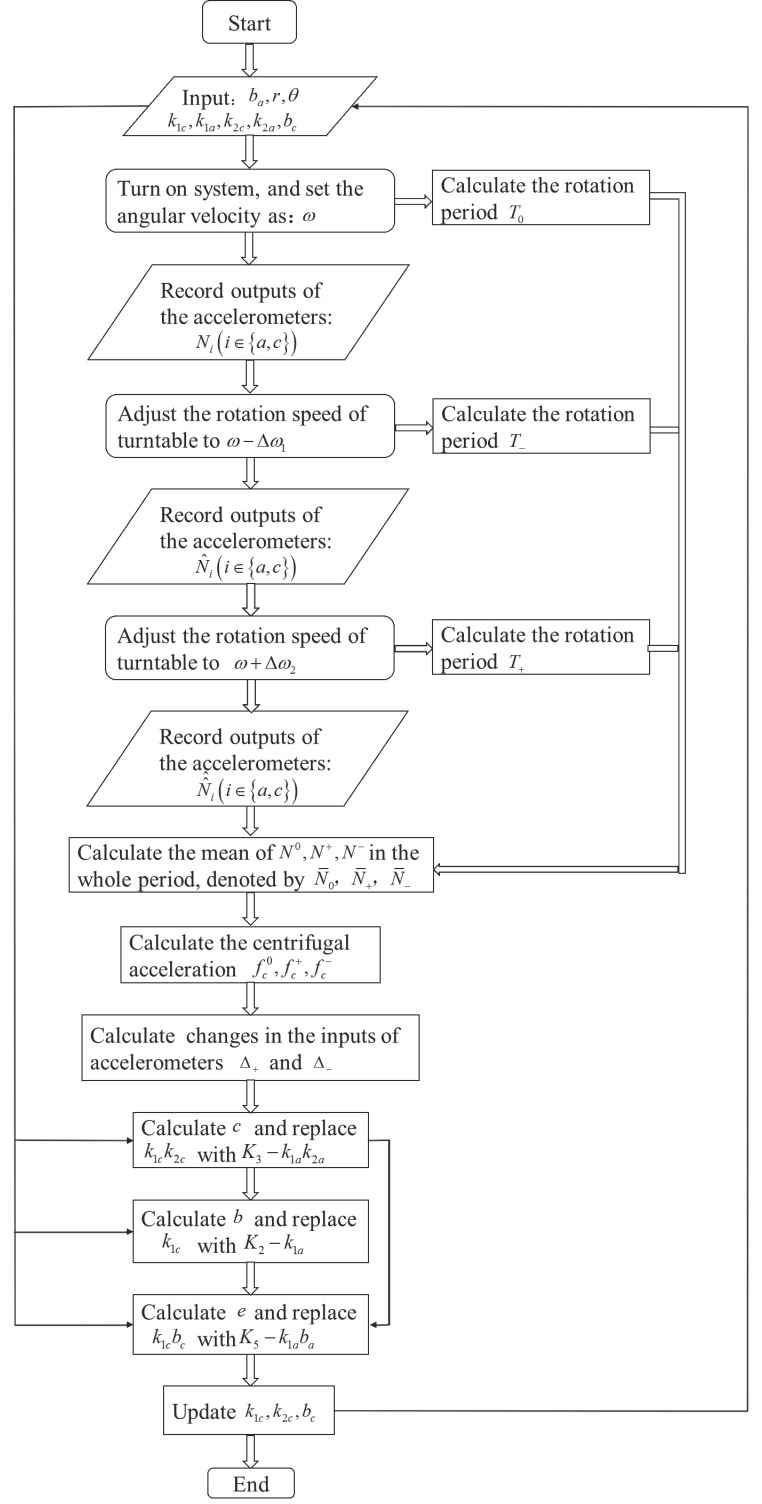
Flow chart of the matching process with the new matching method.

The flow diagram for the new matching method is shown in Figure 4. Next, this method will be presented systematically.

First, parameters $k_{1c},\;k_{1a},\;k_{2c},\;k_{2a},\;b_{c},\;b_{a},\;r$ and θ from Equations (14)–(23) are determined though calibration experiments. The calibration process for the inertial sensor accelerometer includes determining systematic errors comprising of bias, scale factor, and misalignment error coefficients [34,35]. In these calibration experiments, both the mathematical model of the accelerometer with a quadratic factor and high-g acceleration signals are used. Parameters $k_{1c},\;k_{1a},\;k_{2c},\;k_{2a},\;b_{c},\;b_{a},\;r$ and θ are obtained by comparing the excitation acceleration signal and the output responses of the calibrated accelerometer to the excitation signals [35,36]. Kalman filter also plays an important role in the estimating for these parameters [36]. Second, the system is turned on, and the angular velocity of the disc is set to ω. Meanwhile, the outputs of the accelerometers are recorded as $N_{i}\left(i\in \left\{a,c\right\}\right)$. Third, the rotational speed of the disc is adjusted to $\omega -\Delta \omega_{1}$ ($\Delta \omega_{1}$ is a known fixed angle, making the centrifugal acceleration change by $\Delta_{-}$). Similarly, the outputs of the accelerometers are recorded as ${\hat{N}}_{i}\left(i\in \left\{a,c\right\}\right)$. Finally, the turntable speed is reset again to $\omega +\Delta \omega_{2}$ ($\Delta \omega_{2}$ is a known fixed angle, making the centrifugal acceleration change by $\Delta_{+}$), and the outputs of the accelerometers are recorded as ${\hat{\hat{N}}}_{i}\left(i\in \left\{a,c\right\}\right)$.

After the abovementioned data collection, primary data are processed as follows. First, the appropriate data are selected from the records. Here, “appropriate data” refers to the length of data as a multiple integer of the rotation period of the turntable. These data are denoted as ${\tilde{N}}_{i}^{0},{\tilde{N}}_{i}^{+},{\tilde{N}}_{i}^{-},\left(i\in \left\{a,c\right\}\right)$ (the lengths of them are as a multiple integer of the rotation period of the turntable). Then, these data are summarized and the average of the sums are calculated as follows:

(29) $$\begin{array}{l} N^{0}={\tilde{N}}_{a}^{0}+{\tilde{N}}_{c}^{0}\\ N^{+}={\tilde{N}}_{a}^{+}+{\tilde{N}}_{c}^{+}\\ N^{-}={\tilde{N}}_{a}^{-}+{\tilde{N}}_{c}^{-} \end{array}$$

where $N^{0},N^{+},N^{-}$ represent the sum of the accelerometers. For each angular speed of the disc, we calculate the average of $N^{0},N^{+},N^{-}$, denoted by ${\bar{N}}_{0},\;{\bar{N}}_{+},\;{\bar{N}}_{-}$. Finally, with the input of $k_{1c},\;k_{1a},\;k_{2c},\;k_{2a},\;b_{c},\;b_{a},\;r,\;\theta ,\;{\hat{N}}_{0},\;{\hat{N}}_{+},\;{\hat{N}}_{-}$ and the data processing algorithm, the accelerometers are matched.

The whole Update Algorithm is introduced as follows:
**Update Algorithm**Input: $k_{1c},\;k_{1a},\;k_{2c},\;k_{2a},\;b_{c},\;b_{a},\;r,\;\theta ,\;{\hat{N}}_{0},\;{\hat{N}}_{+},\;{\hat{N}}_{-}$.Output: update of $k_{1c},\;k_{2c},\;b_{c}$.1. Calculate the centrifugal acceleration $f_{c}^{0},\;f_{c}^{+},\;f_{c}^{-}$ generated by the rotation of the disc as follows: $f_{c}^{0}=r\omega^{2},\;f_{c}^{+}=r{\left(\omega +\Delta \omega_{2}\right)}^{2},\;f_{c}^{-}=r{\left(\omega -\Delta \omega_{1}\right)}^{2}$.2. Calculate the changes in the inputs of accelerometers, denoted by $\Delta_{+}$ and $\Delta_{-}$; $\Delta_{+}=\left(f_{c}^{+}-f_{c}^{0}\right)\sin\theta$, $\Delta_{-}=\left(f_{c}^{0}-f_{c}^{-}\right)\sin\theta$.3. Calculate $K_{3}=\frac{{\bar{N}}_{-}+{\bar{N}}_{+}-2{\bar{N}}_{0}}{2\Delta_{+}\Delta_{-}}$, and then replace $k_{1c}k_{2c}$ with $K_{3}-k_{1a}k_{2a}$.4. Calculate $K_{2}=\frac{{\bar{N}}_{+}-{\bar{N}}_{-}-2K_{3}F\left(\Delta_{+}+\Delta_{-}\right)}{\Delta_{+}+\Delta_{-}}$, where $F=r\omega^{2}\sin\theta$ and then replace $k_{1c}$ with $K_{2}-k_{1a}$.5. Calculate $K_{5}={\bar{N}}_{0}-K_{2}F-K_{3}F^{2}$, and then replace $k_{1c}b_{c}$ with $K_{5}-k_{1a}b_{a}$.6. Save the updated $k_{1c}k_{2c},\;k_{1c},\;k_{1c}b_{c}$ accelerometer parameters and complete real-time matching.

All the proposed methods can be summarized as follows: First, adjust the rotation speed of the turntable as shown in Figure 4, and collect the data of the accelerometers. Then, data processing is performed according to the data processing algorithm. Finally, the accelerometer is matched by updating the scale factors of accelerometers. Therefore, it can be seen that the new matching method is based on both the geometry of the new design, and the data processing steps. There is an indirect relationship between the relative frequency change Δω/ω and the SNR. And the value of Δω/ω needs to be determined according to different actual situation.

After illustrating the new matching method for accelerometers, we further analyzed the validity of the new matching method via the following simulation experiment.

## 4. Simulation 

A simulation experiment was developed based on the above analysis. The data generated by the simulator were used to validate the new matching method. First, we introduce the design of the simulator for a rotating accelerometer-based gradiometer. Then, the matching performance of the new method is estimated based on the frequency spectrum of the results.

### 4.1. Design and Implementation of Simulation

The parameters of the simulation, including the radius of the disc (r), the inclination of the rotation axis of the disc (ϕ), gravity gradient tensor (Γ, expressed in North-East-Down coordinate system), gravity vector (g, expressed in North-East-Down coordinate system), and the angle between the sensing axis of accelerometer and the tangent line (θ), were set as shown in Table 1.

**Figure 5:**
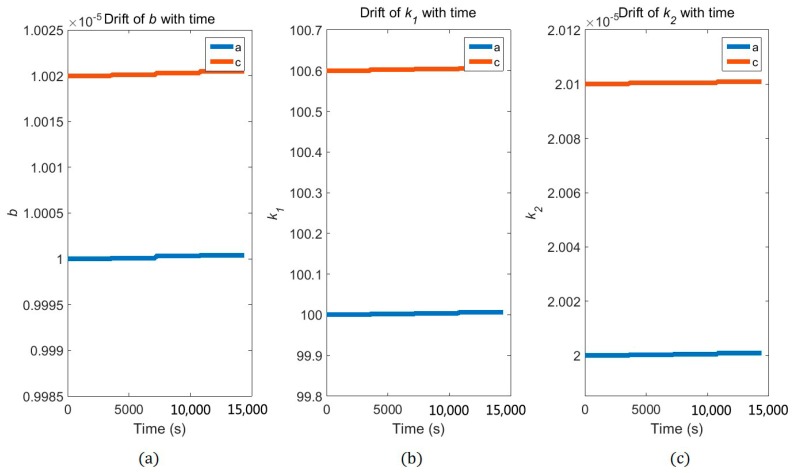
Parameters drift with time for accelerometer *a* and *c*. The variation in the step change for parameters *b*, *k*_1_ and *k*_2_ was shown in the (**a**)–(**c**) panel, respectively. The orange lines represent the accelerometer *a* and the blue lines represent the accelerometer *c*.

For accelerometers in the simulation, the preliminary values of $k_{1a},\;k_{2a},\;b_{a},\;k_{1c},\;k_{2c}$ and $b_{c}$ were set to $100,\;2\times 10^{-5},\;1\times 10^{-5},\;100.1,\;2.004\times 10^{-5}$ and $1.002\times 10^{-5}$, respectively. We assume that these parameters drift with time as shown in Figure 5, which presents the variation in the step change for these parameters. Taking the measurement error of accelerometers into account, the measurement errors were assumed to be white and Gaussian with zero mean and given a standard deviation of $\sigma^{2}=1\times 10^{-7}$.

**Figure 6:**
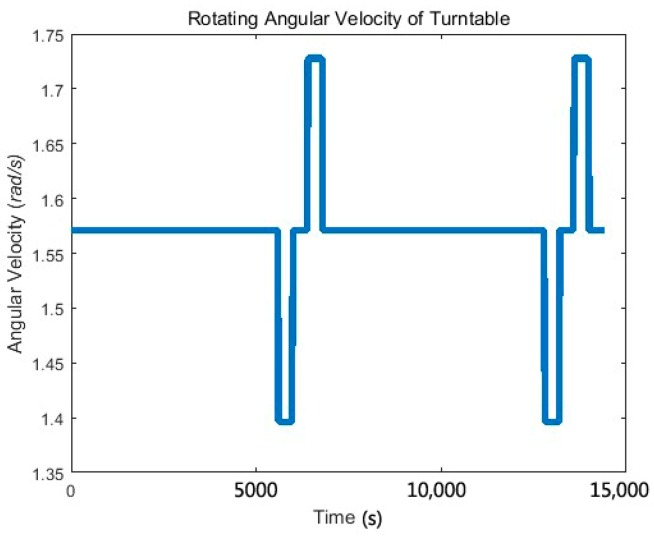
Angular velocity of the disc and its variation with time. The angular velocity of the disc is divided into three stages, and the angular velocity of each stage is denoted as $\omega ,\omega -\Delta \omega_{1}$ and $\omega +\Delta \omega_{2}$.

The angular velocity of the turntable, and its variation with time is shown in Figure 6.

The simulation experiment lasted for two cycles, as shown in Figure 6. However, we only illustrated and analyzed the data in one cycle. During the first 6000 s, the rotational speed of the turntable was ω=π/2 rad/s, and the gradiometer worked in the normal measurement stage. From the 6000th second, the gradiometer entered the matching phase. During the next 400 s, the angular velocity of the turntable was $\omega -\Delta \omega_{1}=4\pi /9\;\mathrm{rad}/s$, i.e., $\Delta \omega_{1}=\pi /18\;\mathrm{rad}/s$. At 6400–6800 s, the angular speed of the disc was ω=π/2 rad/s. At 6800–7200 s, the rotational speed of the turntable was $\omega +\Delta \omega_{2}=11\pi /20\;\mathrm{rad}/s$, i.e., $\Delta \omega_{2}=\pi /20\;\mathrm{rad}/s$. Matching was performed every two hours and lasted for 1200 s.

A set of time series that ’measured’ data of acceleration was obtained through simulation.

### 4.2. Data Processing

**Figure 7:**
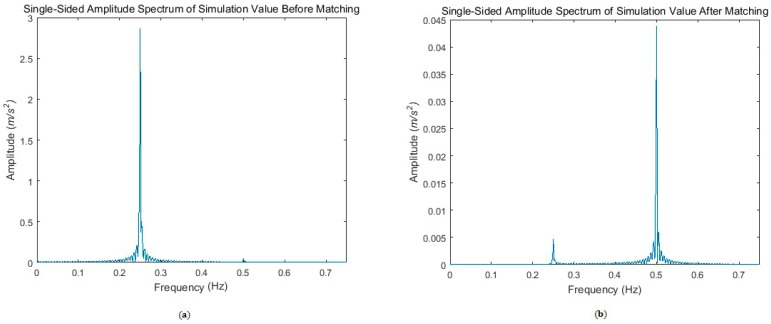
Frequency spectra of $f_{a}+f_{c}$. (**a**) The frequency spectrum of the summation before matching; (**b**) The frequency spectrum of the summation after matching.

The data collected from the simulation experiments were processed in accordance with Section 3.4. The frequency spectrum of pre- and post-processed data are shown in Figure 7.

Figure 7a indicates that most of the energy was concentrated in nearby 0.25 Hz in the frequency domain, which is the rotation frequency of the disc. However, most of the energy of the addition centralized in the frequency at approximately 0.5 Hz (Figure 7b) after matching. Equation (9) reveals that the gravity gradient signals are in a frequency double with the rotation (0.5 Hz). The SNR (signal-to-noise ratio) is defined as follows:

(30) $$\mathrm{SNR}=10\times \log_{10}\left(\frac{\mathrm{Amplitude}\;\mathrm{of}\;\mathrm{signal}\;\left(0.5\;\mathrm{Hz}\right)}{\mathrm{Amplitude}\;\mathrm{of}\;\mathrm{noise}\;\left(\mathrm{others}\right)}\right)$$

Therefore, the result without matching equated to −41 dB. After the accelerometers were matched, the SNR turned to 22 dB. 

## 5. Discussion and Conclusions

The results showed that using the new method suppressed the influence of the mismatch of accelerometer scale factors on measurement. The performance of the new matching method is equivalent to the strategy of trimming bias voltage proposed by Tu et al. [25]. Thus, the validity of the new matching method is acceptable. After using the matching method proposed in this study, the SNR has been significantly improved (from −41 dB to 22 dB), which is a great convenience for the extraction of gravitational gradient signals.

Compared with other methods, no extra hardware support is required for the new matching method, which made the measurement of gravity gradient simpler and cost less. Moreover, the new matching method is achieved by means of updating scale factors based on the matching algorithm. 

Given the close relationship between the matching result and the estimated accuracy of *K_2_*, *K_3_* and *K_5_*, the smaller estimation errors of parameters *K_2_*, *K_3_* and *K_5_* will equate to a higher matching precision of the accelerometer. The estimated values and true values of *K_2_*, *K_3_* and *K_5_* are shown in Table 2.

The estimation error is calculated with the following equation:

(31) $$\mathrm{Estimation}\;\mathrm{error}\;=\;\frac{\mathrm{Estimated}\;\mathrm{value}\;-\mathrm{True}\;\mathrm{value}}{\mathrm{True}\;\mathrm{value}}\times 100\%$$

Table 2 indicates that the estimation error of *K_2_* was 3 × 10^−8^, which is negligible. The estimation error of *K_3_* was −1.9 × 10^−3^, which is an important cause of error for $k_{1c}k_{2c}$. However, the estimation error of *K_5_* was as high as 9.2 × 10^−2^, which will significantly affect the update of $k_{1c}b_{c}$. This result indicates that the performance of the new matching method can still be improved.

There are two key ways to improve matching results. The first is to decrease the interval between matchings, and the second is to improve the estimation performance of the data processing algorithm. 

In conclusion, this new matching method can help decrease the costs of gravity gradient measurement. More importantly, it might provide a new and potential idea for accelerometer matching in the gravity gradiometer. Nevertheless, this proposed method still has some flaws and expected challenges. For example, there is a certain degree of principle error caused by omitting “other second- and higher-order terms” in the mathematical model of the accelerometer with a quadratic factor. Fortunately, the error is so small that it does not affect the results of the matching. The matching method proposed in this paper can improve SNR in gravity gradient measurement with the same accuracy as other methods. However, it is more flexible, convenient and economical. Thus, the new matching method might provide a sound foundation for further research. Furthermore, the accuracy of the new matching method can be improved by modifying the algorithm.

## Figures and Tables

**Table 1:** Simulation parameters.

Parameter	Description	Value and Unit
r	Radius of the turntable	0.2m
ϕ	Tilt angle of the rotating axis	$30^{\circ }$
Γ	Gravity gradient tensor	A
g	Gravity vector (in local frame)	$\left(\begin{array}{c} 0\\ 0\\ 9.8 \end{array}\right)m/s^{2}$
θ	Angle between sensing axis and tangent line	$45^{\circ }$

Where $A=\left[\begin{array}{ccc} -2000 & 1000 & 1000\\ 1000 & -1300 & 1000\\ 1000 & 1000 & 3300 \end{array}\right]\left(E\right)$.

**Table 2:** True values and estimated values of *K_2_*, *K_3_* and *K_5_*.

Parameters	*K_2_*	*K_3_*	*K_5_*
True value	2.006050119 × 10^2^	4.029760031 × 10^−3^	1.839030458 × 10^−3^
Estimated value	2.006050180 × 10^2^	4.022221105 × 10^−3^	2.008082321 × 10^−3^
Estimation error	3.0 × 10^−8^	−1.9 × 10^−3^	9.2 × 10^−2^
